# Isothermal micro calorimetry – a new method for MIC determinations: results for 12 antibiotics and reference strains of *E. coli *and *S. aureus*

**DOI:** 10.1186/1471-2180-9-106

**Published:** 2009-05-26

**Authors:** Ueli von Ah, Dieter Wirz, AU Daniels

**Affiliations:** 1 Research Station Agroscope Liebefeld-Posieux ALP, Bern, Switzerland; 2 Laboratory for Orthopaedic Biomechanics, Clinical Morphology & Biomedical Engineering, University of Basel Faculty of Medicine, Basel, Switzerland

## Abstract

**Background:**

Antimicrobial susceptibility testing of microorganisms is performed by either disc diffusion or broth dilution tests. In clinical use, the tests are often still performed manually although automated systems exist. Most systems, however, are based on turbidometric methods which have well-known drawbacks.

**Results:**

In this study we evaluated isothermal micro calorimetry (IMC) for the determination of minimal inhibitory concentrations (MICs) of 12 antibiotics for five micro-organisms. Here we present the data for the 12 antibiotics and two representative microorganisms *E. coli *(a Gram^-^) and *S. aureus *(a Gram^+^). IMC was able to determine the MICs correctly according to CLSI values. Since MICs require 24 hours, time was not reduced. However, IMC provided new additional data – a continuous record of heat-producing bacterial activity (e.g. growth) in calorimetry ampoules at subinhibitory antibiotic concentrations. Key features of the heatflow (*P*) and aggregate heat (*Q*) vs. time curves were identified (*t*_*delay *_and *ΔQ/Δt *respectively). Antibiotics with similar modes of action proved to have similar effects on *t*_*delay *_and/or *ΔQ/Δt*.

**Conclusion:**

IMC can be a powerful tool for determining the effects of antibiotics on microorganisms in vitro. It easily provides accurate MICs – plus a potential means for analyzing and comparing the modes of action of antibiotics at subinhibitory concentrations. Also IMC is completely passive, so after evaluation, ampoule contents (media, bacteria, etc.) can be analyzed by any other method desired.

## Background

In order to evaluate antimicrobial susceptibility of microorganisms, a variety of methods is available for clinical laboratories [1,2]. The most commonly used are disc diffusion tests or broth dilution tests. For both methods, automated systems exist for determination of the minimal inhibitory concentration (MIC) of an antibiotic for a microorganism and are in use in clinical laboratories [1].

For broth dilution, the automated systems use different methods for detection. They either detect growth or non-growth photometrically, fluorometrically or turbidometrically [1]. One of the most common used systems is the Vitek^® ^or Vitek2^® ^which determines growth turbidometrically at hourly intervals for up to 15 h. Turbidity based methods, however, assume a linear relationship between test organism growth and absorbance [3]. Also, if turbidity is interpreted visually, results can differ from person to person.

All chemical or physical processes either generate or consume heat. This can be measured using isothermal microcalorimetry (IMC). The heat flow rate is proportional to the reaction rate, and the total heat produced in some time t is proportional to the extent of the reaction taking place in time t. Based on these principles, IMC is a universal tool for real-time evaluation of rate processes in small (e.g. 3–20 ml) ampoules, including processes involving cultured cells [4]. In IMC the net heat flow generated by any biological or non-biological chemical or physical processes taking place within the ampoule is continuously measured while the ampoule is kept at constant temperature. IMC instruments can be calibrated with an internal precision heater or with reactions of known heat-flow. However, the instruments measure the net heat flow produced by all processes taking place in an ampoule. Therefore, in order to correctly interpret the measurements, the user must have knowledge of what processes are taking place and have, if necessary, an experimental means for accounting for heat flow from processes not of interest. A prime example is chemical breakdown of the medium in which a process of interest is taking place. Besides being a universal rate process measurement tool, IMC also has the advantage that it is entirely passive. Therefore the specimen is not disturbed in any way during measurement, and after measurement the contents of ampoule can be evaluated by any other means desired. More information is available in a review by Lewis and Daniels (the senior author) giving a detailed description of the nature, advantages and limitations of IMC, including its use in evaluating cellular processes involving bioactive materials [4].

In 1996, the senior author began reporting his experience using isothermal micro-nano calorimetry to evaluate the activity of cultured cells- response of cultured macrophages to implant material particles [5]. However, microcalorimetry has been long-used to study metabolism of cultured cells. James reviewed work in cellular microcalorimetry in 1987 [6] and reported a paper by Hill in 1918 as the earliest employing microcalorimetry to study bacteria. In 1977, Ripa *et al*. [7] evaluated microcalorimetry as tool for the evaluation of blood culture media. In the study, the influence of additives on blood culture could be determined much faster and easier compared to traditional media evaluation methods. Based on their data, Ripa *et al*. [7] suggested the use of microcalorimetry as tool to evaluate the inhibitory or stimulatory influence of various compounds.

Later, another study used microcalorimetry to detect the growth of microorganisms [8]. Other studies described the use of microcalorimetry to evaluate the antimicrobial actions of propiolis extracts [9] or selenium on bacterial strains [10]. Antoce *et al*. [11] successfully used calorimetric methods for the determination of inhibitory effects of alcohols on yeasts to avoid computational errors based on direct assessment of bioactivity using turbidity. An important feature of this method was first noted in the study of Garedew *et al*. [12]: microcalorimetry can provide rapid detection of bacterial growth. If the number of bacteria in a calorimeter ampoule rise to about 10^4 ^cfu they can be detected by their heat production. If growth continues, the heat flow rate will continue to rise for some time.

This was used to advantage in our laboratory in a recently published study in which we employed isothermal microcalorimetry for rapid detection of MSSA and other microorganisms in blood products, i.e. platelet concentrates [13]. Still more recently, we also successfully determined the MIC of cefoxitin for a MRSA strain and a MSSA strain [14]. However, IMC did not decrease the time for MIC determination because MICs are based on detection of growth at 24 hours. But more importantly, IMC with media containing added antibiotic concentrations provided a means for rapidly differentiating between MRSA and MSSA. In addition, it was apparent that the nature of the heatflow curves at subinhibitory concentrations of the antibiotic might provide new insights into the way in which antibiotics affect growth rates. Therefore, we conceived this study.

To further evaluate IMC we have now determined the MICs of 12 antibiotics for reference strains of five organisms, *E. coli *ATCC25922, *S. aureus *ATCC29213, *Pseudomonas aeruginosa *ATCC27853, *Enterococcus faecalis *ATCC29212, and *Streptococcus agalactiae *ATCC27956. In the interest of brevity we report here only the results for *E. coli *ATCC25922 and *S. aureus *ATCC29213 as representatives for Gram^- ^and Gram^+ ^bacteria, respectively.

## Results

**Figure 1:**
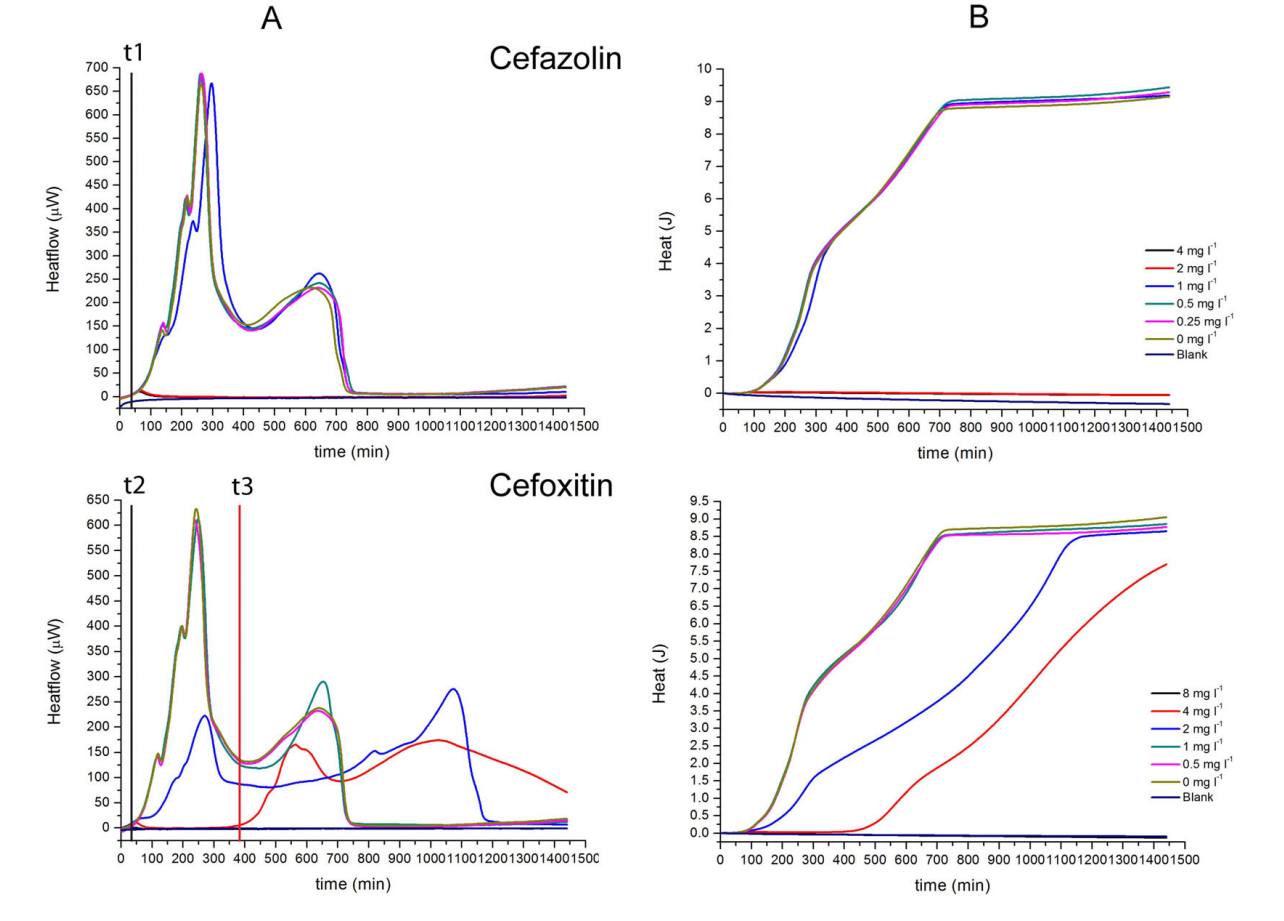
**Heatflow data (column A) and resultant cumulative heat curves (column B) for IMC determinations of the MICs of the respective cephalosporines for *E. coli *ATCC25922 incubated at 37°C**. Culture medium was cation-adjusted Mueller-Hinton II broth. t1, t2: *t*_*delay *_for 0 mg l^-1 ^antibiotic; t3: *t*_*delay *_for 4 mg l^-1 ^cefoxitin. Blank is medium alone. Curves are the mean of three replicates.

**Figure 2:**
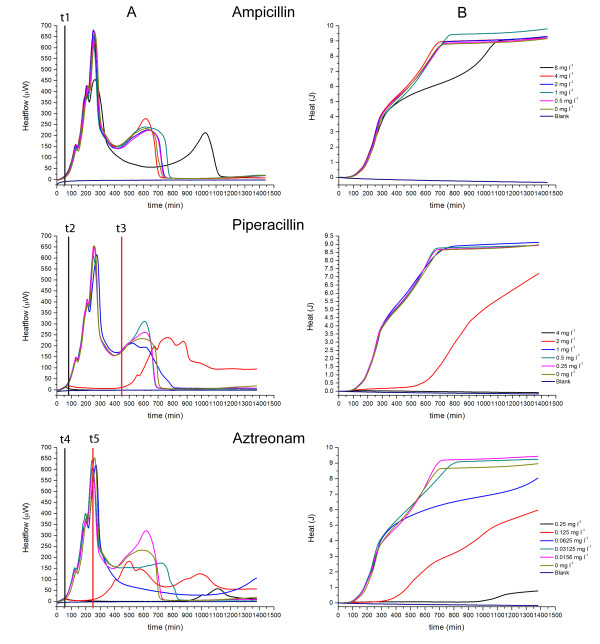
**Heatflow data (column A) and resultant cumulative heat curves (column B) for the IMC determinations of the MICs of ampicillin, piperacillin and aztreonam for *E. coli *ATCC25922 using IMC**. Experiments were performed in cation-adjusted Mueller-Hinton II broth at 37°C. t1, t2, t4: *t*_*delay *_for 0 mg l^-1 ^antibiotic; t3: t_*delay *_for 2 mg l^-1 ^piperacillin; t5: *t*_*delay *_for 0.125 mg l^-1 ^aztreonam. Blank is medium alone. Curves are the mean of three replicates.

**Figure 3:**
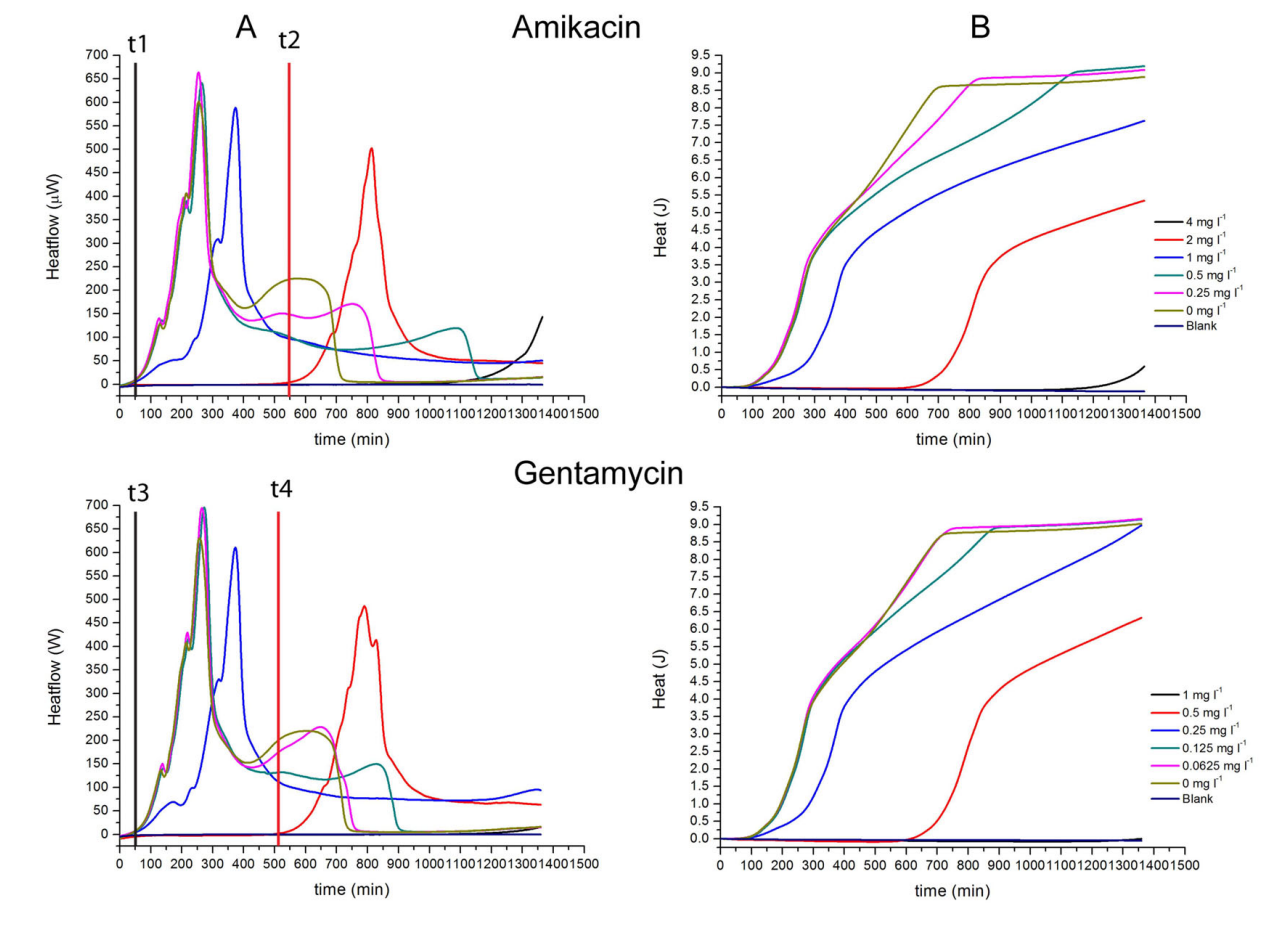
**Heatflow data (column A) and resultant cumulative heat curves (column B) for the IMC determinations of the MICs of amikacin and gentamycin for *E. coli *ATCC25922 in cation-adjusted Mueller-Hinton II broth incubated at 37°C**. t1, t3: *t*_delay _for 0 mg l^-1 ^antibiotic; t2: *t*_*delay *_for 2 mg l^-1 ^amikacin; t4: *t*_*delay *_for 0.5 mg l^-1 ^gentamycin. Blank is medium alone. Curves are the mean of three replicates.

**Figure 4:**
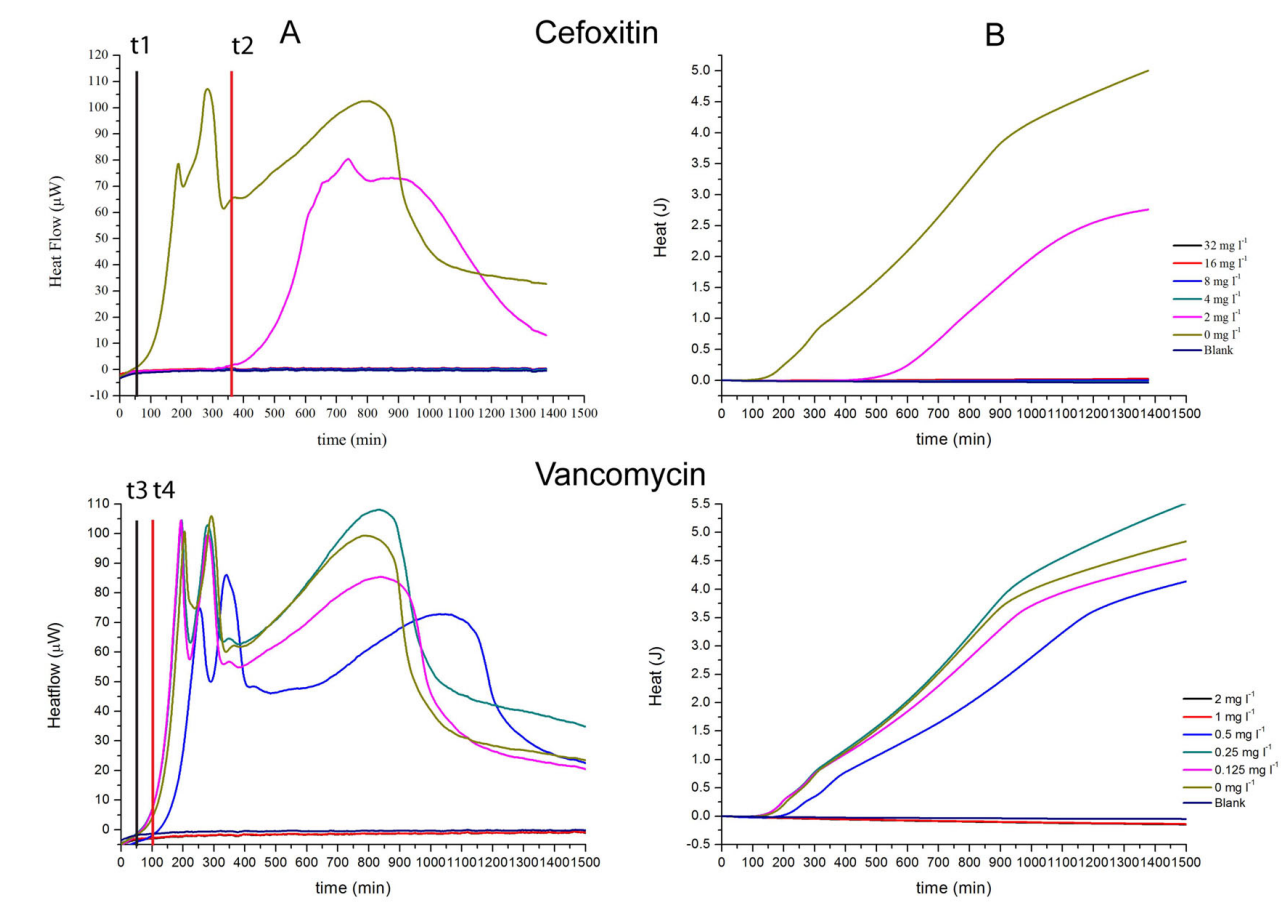
**Heatflow data (column A) and resultant cumulative heat curves (column B) for the IMC determinations of the MICs of cefoxitin and vancomycin for *S. aureus *ATCC29213**. Cultures were incubated at 37°C in cation-adjusted Mueller-Hinton II broth. t1, t3: *t*_*delay *_for 0 mg l^-1 ^antibiotic; t2: *t*_*delay *_for 16 mg l^-1 ^cefoxitin; t4: *t*_*delay *_for 0.5 mg l^-1 ^vancomycin. Blank is medium alone. Curves are the mean of three replicates.

**Figure 5:**
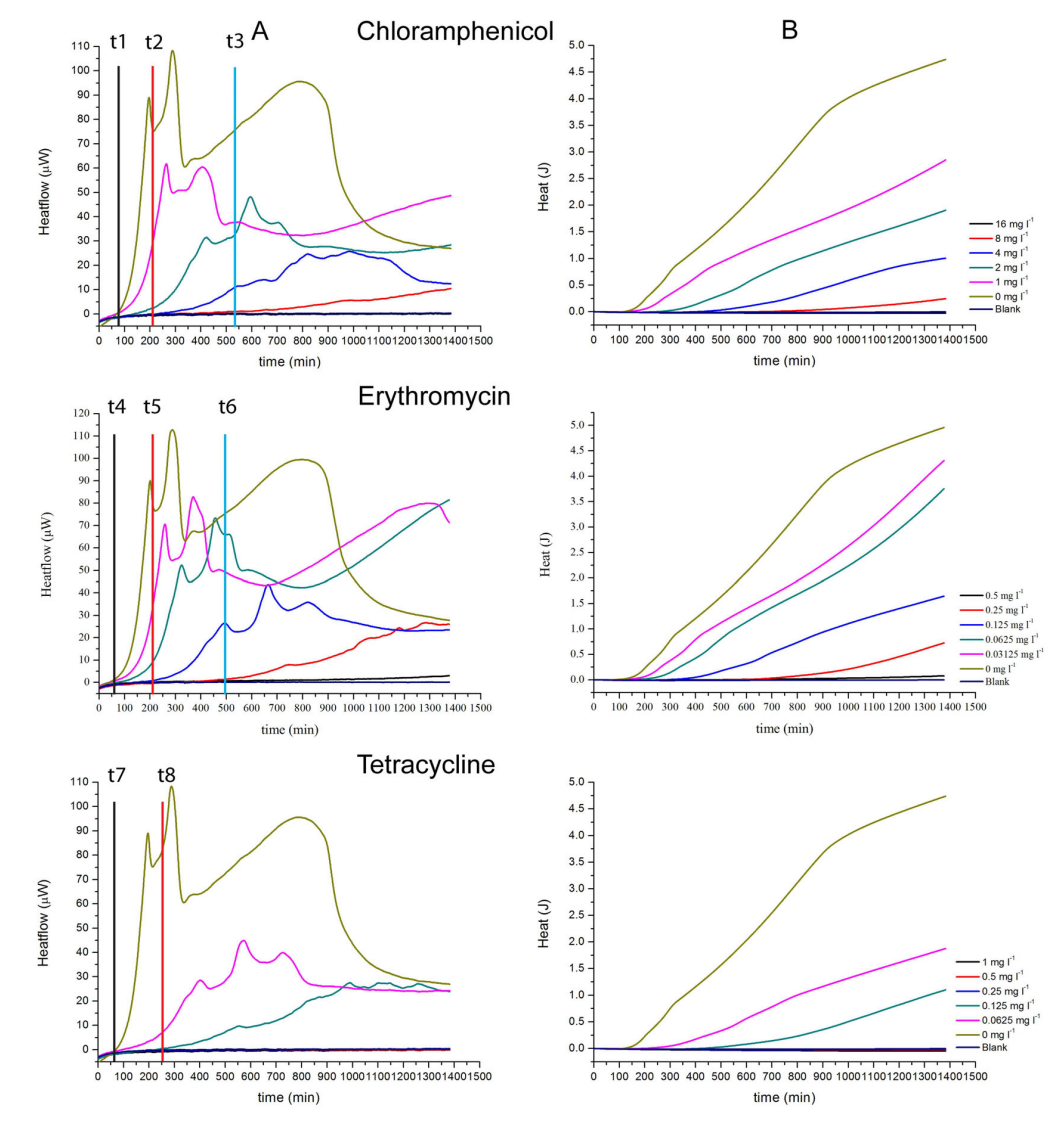
**Heatflow data (column A) and resultant cumulative heat curves (column B) for the IMC determinations of the MICs of chloramphenicol, erythromycin and tetracycline for *S. aureus *ATCC29213**. Experiments performed in cation-adjusted Mueller Hinton II broth at 37°C. t1, t4, t7: *t*_*delay *_for 0 mg l^-1 ^antibiotic; t2: *t*_*delay *_for 4 mg l^-1^, t3: *t*_*delay *_for 8 mg l^-1 ^chloramphenicol; t5: *t*_*delay *_for 0.125 mg l^-1^, t6: *t*_*delay *_for 0.25 mg l^-1 ^erythromycin; t8: *t*_*delay *_for 0.125 mg ^-1 ^tetracycline. Blank is medium alone. Curves are the mean of three replicates.

**Figure 6:**
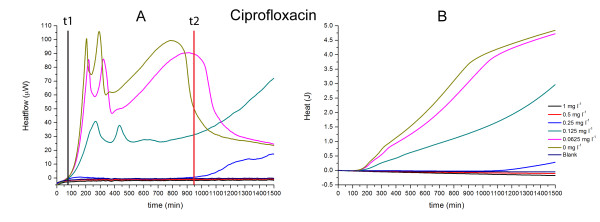
**Heatflow data (column A) and resultant cumulative heat curves (column B) for the IMC determinations of the MICs of ciprofloxacin for *S. aureus *ATCC29213 in cation-adjusted Mueller-Hinton II broth incubated at 37°C**. t1: *t*_*delay *_for 0 mg l^-1 ^antibiotic; t2: *t*_*delay *_for 0.25 mg l^-1 ^ciprofloxacin. Blank is medium alone. Curves are mean of three replicates.

As is evident in Figs. 1, 2, 3, 4, 5 and 6, the heat flow rate signals from blank ampoules (no inoculum) never departed appreciably from baseline over the time of measurement. That is, the blanks produced no appreciable heat flow – especially compared to the peak values (often > 100 μW) measured when bacteria were present. Thus all heat flow signals above baseline could be attributed to bacterial activity and growth. Table 1 provides an overview comparing the MICs determined by IMC with those determined by a standard turbidometric method. It also provides a comparison of key growth-related calorimetric parameters determined at subinhibitory concentrations just below the MIC value: *t*_*delay *_(delay in time of onset of detectable heat flow), and *P*_*max *_(maximum rate of heat production). These and other calorimetric parameters pertinent to this study and derived from the data are explained and used in the Discussion section.

**Table 1:** Overview of the comparison of the broth dilution method as described by the CLSI [15] and the IMC method developed in this study.

	MIC (CLSI) [mg l^-1^]	MIC (IMC) [mg l^-1^]	*t*_*delay *_[min]	*P*_*max *_[μW]
***E. coli***				
Cefazoline	2	2	54	666
Cefoxitin	8	8	402	174
Ampicillin	n. d.^a^	n. d.	0	454
Piperacillin	4	4	445	237
Aztreonam	n. d.	n. d.	950	57
Amikacin	n. d.	n. d.	1145	o. t.^b^
Gentamycin	1	1	560	486
***S. aureus***				
Cefoxitin	4	4	412	80
Vancomycin	1	1	147	339
Chloramphenicol	16	16	630	o. t.
Erythromycin	0.5	0.5	638	27
Tetracycline	0.25	0.25	330	27
Ciprofloxacin	0.5	0.5	1097	17 (almost o. t.)

*t*_*delay *_and *P*_*max *_show the values of the curves determined at one concentration below the MIC value.

^a ^n. d.: not determinable using the tested concentrations.

^b ^o. t.: Outside measuring time.

### MICs for *E. coli *ATCC25922

We evaluated the MICs of 12 different antibiotics for *E. coli*. For brevity, we present here the results for 7 antibiotics grouped by mode of action. The antibiotics used and their concentrations can be found in the corresponding figures. All evaluations were also performed in parallel using the standard method – visual detection of turbidity at 24 hours. Unless otherwise stated, the results for the MIC determination were the same for calorimetry and the standard visual method. In Figs. 1, 2, 3, 4, 5 and 6, Column A shows the recorded heat flow rate data (μW = μJ/s vs. time in min.). Any time delay (*t*_*delay*_) before a heat signal was recorded was the time required until there were sufficient numbers of active bacteria to produce a heat flow signal above the instrument's detection limit. The highest peak in a μW vs. time curve indicates the maximum rate of heat production observed (*P*_*max*_). Column B presents the results of integrating the data in Column A to show the cumulative amount of heat produced over time (J vs. time in min.). As explained later, the Column B curves are somewhat analogous to conventional growth curves showing the increase in the number of bacteria over time. Mean slopes (*ΔQ/Δt*) for a given portion of an aggregate heat curve are aggregate rates of heat production and indicative of their rate of bacterial growth. Maximum values (*Q*_*max*_) are related to the total numbers of cells produced by time t.

*E. coli *and cephalosporines of the 1^st ^and 2^nd ^generation. (Fig. 1). The 1^st ^generation cephalosporine used in this study was cefazolin and its MIC for *E. coli *was correctly determined using IMC as 2 mg l^-1 ^based on the recommendations of the CLSI [15]. At the MIC and higher concentrations there was essentially no growth. However, there was a slight temporary increase in heatflow at the beginning of the experiments. This suggests a slight transitory increase in metabolic activity of the bacteria present, followed by no subsequent growth. At all subinhibitory concentrations, heat production of *E. coli *was the same (same *t*_*delay*_, *P*_*max*_, *ΔQ/Δt*, and *Q*_*max*_).

Cefoxitin was used as an antibiotic representing the 2^nd ^generation of cephalosporines although it is a member of a subgroup of this generation and also active for anaerobic bacteria. The cefoxitin MIC could also be determined correctly using IMC as 8 mg l^-1^. In contrast to cefazolin, there was no transient initial increase in heatflow at the MIC (Fig. 1A).

Also, the profiles of the curves at subinhibitory concentrations differed markedly between cefazolin and cefoxitin (Fig. 1). For cefoxitin, *t*_*delay *_(Fig. 1A) increased with increasing concentration, and antibiotic tended to lower *P*_*max*_. Also initial *ΔQ/Δt *values (Fig. 1B) declined with increasing antibiotic concentration, but *Q*_*max *_tended to a maximum value (~9 J) independent of antibiotic concentration. The calorimetric method thus highlighted differences in action of the two cephalosporines.

*E. coli *and penicillins. (Fig. 2). Ampicillin and piperacillin were tested as members of the penicillin family. Additionally, the monobactam aztreonam was included in this group, because it is another antibiotic interacting with cell wall synthesis but with a different mode of action. The grouping with ampicillin and piperacillin also facilitated a comparison of the curve profile differences. For ampicillin, the MIC could not be determined by either method with the range of concentrations used, although a decrease in heatflow could be detected for 8 mg l^-1^. For piperacillin, the MIC for *E. coli *was determined as 4 mg l^-1 ^which corresponds to the value for quality control in the CLSI manual [15]. At the beginning of the experiment, a slight transient increase of the heatflow curve was detected at the MIC as well as on the delayed heatflow curve for a concentration of 2 mg l^-1 ^piperacillin (Fig. 2). The MIC for aztreonam was "on the edge" of determination as 0.25 mg l^-1 ^using standard methods (OD_600 _0.06). However, the results of IMC show that the MIC was higher, and the tested concentrations were too low (Fig. 2).

As discussed above, the concentrations of ampicillin were too low to provide much information. However, at 8 mg l^-1 ^*P*_*max*_decreased. The profiles of the heatflow curves were similar for piperacillin and aztreonam and (Fig. 2A). The heatflow curve at the highest subinhibitory concentration of aztreonam (0.25 mg l^-1^) had a higher *t*_*delay *_than the one for piperacillin (2 mg l^-1^) – roughly 950 min vs. 445 min. As is generally the case, antibiotics tended to lower *P*_*max*_. For the heat curves (Fig. 2B) the initial *ΔQ/Δt *values declined with increasing antibiotic concentration, but the effect was stronger for aztreonam. As before, *Q*_*max *_values tended toward a maximum of 9–10 J not related to antibiotic concentration.

*E. coli *and bacterial protein synthesis inhibitors. (Fig 3.) Two antibiotics inhibiting bacterial protein synthesis were evaluated, amikacin and gentamycin. For gentamycin, the MIC was determined as 1 mg l^-1 ^which is in concordance with the reference MIC as proposed by the CLSI manual [15]. For amikacin, the MIC could not be determined with the tested concentration range by either method. For IMC, after approx. 1100 min (~18 hours) the heatflow curve of the highest concentration of 4 mg l^-1 ^started to increase. The growth of *E. coli *at this concentration was also confirmed using the standard method, resulting in an OD_600 _of 0.2 for the samples in the calorimeter and 0.7 for the samples in the water bath.

The profiles of the heatflow and heat curves at subinhibitory concentrations, however, were similar for both antibiotics: For heatflow (Fig. 3A) *t*_*delay *_increased with increasing concentration, and *P*_*max *_decreased. For heat (Fig. 3B), there was virtually no effect on *ΔQ/Δt*. As before, *Q*_*max *_tended to a value of 9–10 J independent of whether an antibiotic was present. The calorimetric data thus suggest the modes of action of amikacin and gentamycin on *E. coli *are essentially the same.

### MICs for *S. aureus *ATCC29213

For *S. aureus *we determined the MICs of 10 of the 12 antibiotics using IMC. However, for the sake of brevity and to illustrate the main findings, we only present 6 antibiotics which have the same modes of action as those presented for *E. coli*. The tests were also performed in parallel in a water bath and evaluated using the standard visual turbidity method. Again, unless otherwise stated, the results of both methods were in agreement with each other.

*S. aureus *and cell wall synthesis inhibitors. (Fig. 4) The antibiotics evaluated were cefoxitin and vancomycin. For cefoxitin, the MIC was determined as 4 mg l^-1 ^whereas vancomycin had a MIC of 1 mg l^-1^. Both values were in agreement with the reference MIC in the CLSI manual [15].

For both antibiotics, the *t*_*delay *_values for the heatflow curves (Fig. 4A) increased with increasing concentration, but the effect was stronger for cefoxitin. Also *P*_*max *_was reduced at the highest concentration not inhibiting growth. For the heat curves (Fig. 4B) there was little change in *ΔQ/Δt *with antibiotic concentration. However *Q*_*max*_declined, but as shown, the highest value observed was ~5 J. This is far below the maximum value of 9–10 J seen repeatedly for *E. coli*, independent of antibiotic concentration, and differences here can be attributed to differences in *t*_*delay*_. Thus the chief difference shown by IMC was the stronger effect of cefoxitin on initial bacterial activity.

*S. aureus *and protein synthesis inhibitors. (Fig. 5) The MICs were determined as 16 mg l^-1^, 0.5 mg l^-1 ^and 0.25 mg l^-1 ^for chloramphenicol, erythromycin and tetracycline, respectively, which are identical to the values in the CLSI manual [15]. The overall profiles of the subinhibitory heatflow curves (Fig. 5, column A) and heat curves (Fig. 5, column B) were remarkably similar for all three antibiotics. None of the three antibiotics produced a substantial increase in *t*_*delay*_. The only substantial difference was for the maximum heatflow rate, *P*_*max*_. Tetracycline had a much larger influence on *P*_*max *_than the other two antibiotics. All three antibiotics produced a decline in *ΔQ/Δt *with increasing concentration. Changes in *Q*_*max *_with concentration can be attributed to the differences in *ΔQ/Δt*. The IMC data suggest that all three antibiotics affect the rate of bacterial growth but do not delay its onset.

*S. aureus *and an antibiotic acting on DNA. (Fig. 6) Only one antibiotic was tested which interacts with bacterial DNA, namely ciprofloxacin. The MIC was determined as 0.5 mg l^-1 ^using IMC which corresponds to the reference value in the CLSI manual [15].

For ciprofloxacin, there was no increasing *t*_*delay*_with increasing concentration (Fig. 6A) except for the concentration one level below the MIC. However, the maximum heatflow rate *P*_*max *_decreased with increasing concentration. For aggregate heat (Fig. 6B) *ΔQ/Δt *declined with increasing concentration. The effect of ciprofloxacin concentration on *Q*_*max *_can be attributed almost entirely to its effect on growth rates. In summary, IMC data suggest that ciprofloxacin delayed onset of bacterial growth somewhat but its principle action was to decrease the rate of subsequent growth.

## Discussion

In this paper, we present results for the use of isothermal microcalorimetry (IMC) as tool for the determination of the minimal inhibitory concentration (MIC) of different antibiotics on *Escherichia coli *ATCC25922 and *Staphylococcus aureus *ATCC29213 and the effects of subinhibitory concentrations on the nature of growth. We have already shown previously that IMC allows the differentiation of MRSA from MSSA [14], and Antoce *et al*. used IMC to determine the inhibitory effect of C1-C4 *n*-alcohols on the growth of yeast species [11]. The same group concluded that if the heatflow curves of the calorimetric measurement are delayed and no change in slope could be determined, the inhibitory compound is only bacteriostatic – acting by reducing the initial bacterial cell count. A 1978 study by Semenitz [16] measured the MIC's of oleandomycin and erythromycin against *S. aureus*. He used an early "flow calorimeter" and its resolution was not at the same level as the sealed-ampoule calorimeters used in this study. He also mistook suppression of a second growth peak as evidence of the determination of an MIC.

Cases in which MICs were not determined. In some of our experiments shown here, we were not able to determine the MIC value. Nevertheless, we included those results in this study to show that even if the MIC would be higher than the tested concentrations, IMC allows conclusions on the mode of action of antibiotics and to a certain extent an estimation on the MIC. For amikacin, for example, the MIC was higher than the tested concentrations in this study (Fig. 3). However, at a concentration of 4 mg l^-1 ^amikacin, growth started only after approximately 1080 min. Therefore one can estimate that 8 mg l^-1 ^amikacin would produce no growth in 24 hours and would thus be the MIC in this case.

We suggest that the reason why the MIC could not, in some cases, be determined in accord with the CLSI manual was not due to use of IMC but rather due to the preparation of the samples. First, we found no discrepancies between results for IMC and the standard turbidity method. Furthermore, according to the CLSI manual, causes for differing MICs can include altered activity of the antibiotics solution, change in inoculum activity or size, and culture environment factors [15]. In the case of amikacin, it was most likely a reduced activity of the antibiotic due to wrong handling during delivery (uncooled). We also had no results by either method for another bacterium tested (data not shown).

Key features of IMC data at subinhibitory concentrations of antibiotics. For subinhibitory concentrations of antibiotics, IMC provides a detailed record of heat production related to bacterial activity including growth. The heat flow and heat curves show that heat-producing activity is far from constant, and suggest that the curves are potential "signatures" for a given bacteria, growth medium and antibiotic that also may help us understand antibiotic modes of action. The following key features of the heatflow (*P *vs. *t*) and aggregate heat (*Q *vs. *t*) curves are used in the subsequent discussion of our results:

Delay in time of onset of detectable heat flow. (*t*_*delay*_) Detectable heat flow means there are a sufficient number of active bacteria to produce a heat signal above the instrument's detection limit. If the initial number of bacteria present does not produce detectable heat, then subsequent detection of a heat signal essentially constitutes detection of increased bacterial activity potentially including growth. For the initial bacterial concentrations used here, some bacteria exhibit a *t*_*delay *_which is a function of antibiotic concentration. A clear example of an antibiotic producing a *t*_*delay *_alone is the effect of Cefoxitin on *E. coli*. The effect can be seen in either the heat flow rate (Fig. 1A) or cumulative heat data (Fig. 1B). Agents which produce delays in onset of growth are generally termed "bacteriostatic." Thus for a given growth environment and initial bacterial concentration, *t*_*delay *_values could be used to compare levels of bacteriostatic activity.

Maximum rate of heat production (*P*_*max*_). In all examples presented here, a transient maximum rate of heat production *P*_*max *_was observed. In many of the examples, the magnitude of *P*_*max *_declined as a function of increasing subinhibitory antibiotic concentration. The effect of Amikacin on *E. coli *is a clear example (Fig. 3A), as is the effect of Chloramphenicol on *S. aureus *(Fig. 5A). In some cases there was also a substantial second transient maximum of lower value (See Fig. 1A, *E. coli *and Cefazolin and Fig. 4A, *S. aureus *and Vancomycin). The value *P*_*max *_is the aggregate rate of heat production of all bacteria present at the time when the maximum occurs. It depends on both the number of active bacteria present at that time, and the rate at which each bacteria present is producing heat at that time. A separate measurement of the number of bacteria present would be needed in order to use the result to determine the mean heat production per bacterium at the time of the maximum. So while the "P max effect" is interesting as part of the "signature" of the thermodynamic response of bacteria to antibiotics, it is not possible to tell whether the antibiotic is affecting the number of bacteria present, their mean rate of heat production or both. One possibility is that *P*_*max *_peaks are due at least in part to "energy spilling" – transient production of "excess" heat in processes other than growth or maintenance of cell viability [17].

Rate of aggregate heat production. (*ΔQ/Δt*). In preliminary studies (data not shown) we have found that in general the aggregate heat Q at any time t is related to the number of bacteria present, and thus that the change *ΔQ/Δt *for a given portion of the *Q *vs. *t *data is roughly proportional to the rate of bacterial growth during the time *Δt*. A clear example of an antibiotic producing change in *ΔQ/Δt *alone as a function of antibiotic concentration is the effect of Chloramphenicol on *S. aureus *at times up to ~900 minutes (Fig. 5B). Antibiotics which change *ΔQ/Δt *as a function of their concentration could be called "growth rate inhibitors."

Maximum aggregate heat *Q *at time *t*. (*Q*_*max*_) Fig. 5B (*S. aureus*, Chloramphenicol) also provides a clear example of this key feature. In this case differences in *Q*_*max*_as a function of concentration are clearly related to differences in growth rate as measured by *ΔQ/Δt*. However, our IMC method employs sealed ampoules which thus have fixed initial amounts and types of liquid medium and gas mix in the headspace, fixed total volume, and no means of removing products of bacterial activity. Thus there is a limit to the amount of heatproducing bacterial activity (including growth) which can take place. Therefore if sufficient time elapses, the *P*_*max *_values tend back toward baseline and the related *Q*_*max *_values tend to reach the same maximum value for all subinhibitory antibiotic concentrations of a given antibiotic. This is clearly seen for *S. aureus *and Cefazolin (Fig. 1, Column B). Looking at the data in Fig. 5 for *S. aureus *alone (i.e., 0 mg l^-1 ^Choramphenicol) one can see that at about 900 minutes, aggregate heat production *Q *is slowing and starting to approach a maximum.

Therefore, we conclude that the value of *Q *at any time *t *depends on whether the bacteria are still active or whether activity is either becoming increasingly limited by the sealed-system environment or has finally ceased. In fact, our results suggest that the ultimate value of *Q*_*max *_is strictly related to the closed system used and is not different for different antibiotics. Figs 1, 2 and 3 show data for 7 different antibiotics for *E. coli*. All exhibit maximum values of *Q*, and the values were all approximately 9–10 J, regardless of which antibiotic was employed. Thus it does not appear that *Q*_*max *_provides much information regarding antibiotic effects – except as another way to express the information contained in *ΔQ/Δt *at a given place in the time history.

Using IMC data to compare modes of action. By using the above key features of all heatflow and aggregate heat curves of the antibiotics for a single bacterium, it is possible to quite an extent to group the antibiotics by their modes of action. This is best illustrated by examining the results for *S. aureus *(Fig. 4, 5 and 6). Effects on growth for antibiotics interacting with cell wall synthesis (Fig. 4) were completely different from those interacting with protein synthesis (Fig. 5) and DNA synthesis (Fig. 6). Within those groups, there were also slight differences in the curves which are most likely related to the power of the antibiotic against the tested strain or a different interaction site.

Cell wall synthesis inhibitors (Fig. 4) seemed to have mainly a bacteriostatic effect on *S. aureus*. Onset of detectable growth-related activity was delayed, but the subsequent rate was little affected by antibiotic concentration. This was especially evident for cefoxitin. The antibiotics interacting with cell wall synthesis of *S. aureus *delay onset of detectable activity (increase *t*_*delay*_) and reduce the maximum rate of heat-producing activity (*P*_*max*_), but they don't change the subsequent rate of increase (*ΔQ/Δt*) curves (rate of growth). So any reduction in the maximum amount of activity (*Q*_*max*_) that has occurred by a given time is due to *t*_*delay*_.

The difference in the mode of action of the two antibiotics can also be seen. Vancomycin has a unique mode of action inhibiting the second stage of cell wall synthesis whereas cefoxitin has the same mode of action as beta-lactam antibiotics such as penicillins [18-20]. The *t*_*delay *_with vancomycin was much shorter for the concentration just below the MIC than for cefoxitin (Fig. 4A). For cefoxitin, the concentration range was too high. The highest concentration should have been 2 mg l^-1^. However, based on the data for vancomycin and for cefoxitin on *E. coli *(Fig. 1), it can be supposed that *t*_*delay *_would again decrease with decreasing concentrations of cefoxitin. This assumption is also strengthened by our results for other bacteria with cefoxitin (data not shown). Further investigation would make it clear whether antibiotics inhibiting transpeptidases and carboxpeptidases such as cefoxitin have a stronger effect than those interacting with the cell wall peptidoglycans [20].

In contrast, antibiotics related to protein synthesis in *S. aureus *(Fig. 5A) both delayed the onset of detectable growth and reduced the subsequent growth rate as a function of concentration. Tetracycline, which acts on the 30S ribosome by inhibition of the delivery of charged tRNA molecules [20], showed a stronger inhibition than either erythromycin or chloramphenicol, as the decrease was much greater. On the other hand, erythromycin was less strong than chloramphenicol. Both act on the 50S ribosome but on different sites.

Erythromycin acts on the association of peptidyl-tRNA with the P-site whereas chloramphenicol inhibits the peptidyltransferase [20]. These results suggest that IMC might be a powerful tool to evaluate differences in the potency of changes in antibiotic concentration for antibiotics acting against protein synthesis. However, further studies would be needed to validate this suggestion.

In this study, we only tested one antibiotic interacting with DNA synthesis for *S. aureus *(Ciprofloxacin, Fig. 6). Therefore, it's not possible to generalize on an IMC profile characteristic of this group of antibiotics. However, based on the experiments above, there are strong indications that this would be possible. As described above, ciprofloxacin, as a member of this group, has a large effect on *P*_*max *_but only slightly reduces *ΔQ/Δt *(Fig. 6). However, 0.25 mg l^-1 ^ciprofloxacin, which is one dilution above the MIC, had a more dramatic effect on the growth of *S. aureus *than other antibiotics with the same level of dilution tested. This might be related to the mode of action of ciprofloxacin which is inhibition of the gyrasecatalysed super coiling [20,21].

The antibiotics interacting with the cell wall synthesis of *E. coli *could be grouped into three groups based on their heatflow curve profile which, however, were not related to the class of antibiotics (Fig. 1 and Fig. 2).

It was possible to differentiate classic cephalosporines from 2^nd ^generation cephalosporines based on their profile (Fig. 1) although both have the same working mechanism [20]. Subinhibitory concentrations of cefazolin had almost no effect on the heatflow curves compared to cefoxitin (Fig. 1A). It would be interesting to see, whether a 3^rd ^generation cephalosporine has as well another profile.

By comparing the IMC curves of cefoxitin with *E. coli *(Fig. 1) and *S. aureus *(Fig. 4) it can as well be seen that the profile is different for different bacterial species. In this case, it is even more evident since the cell wall is built up differently for *E. coli *(Gram- bacterium) and *S. aureus *(Gram^+ ^bacterium). However, the same effect can be seen on other bacteria of the same type of (data not shown).

Interestingly, the heatflow profiles for piperacillin and aztreonam were very similar (Fig. 2). However, piperacillin had a stronger inhibitory effect on *E. coli *growth than aztreonam. In contrast to other antibiotics sharing the same heatflow profile, the heat curves of *E. coli *incubated with aztreonam or piperacillin were different. It seems that aztreonam has as well an effect on the growth rate at a later stage during incubation (Fig. 2B). This correlates partly with the heat curves of *E. coli *with cefoxitin (Fig. 1B). According to Georgopapadakou *et al*. [22] aztreonam has a similar mode of action as cephalosporines which would explain the similarity in the heat curves.

According to the IMC results, the MIC of aztreonam for *E. coli *was higher than 0.25 mg l^-1^. This was somewhat confirmed by measuring an OD_600 _value of 0.05 at the end of incubation. By visual interpretation, the MIC would have been chosen as 0.25 mg l^-1^. It seems that the slight increase in the heatflow curve of *E. coli *with 0.25 mg l^-1 ^aztreonam after 950 min might be related to transient initial growth-related activity of the bacteria before aztreonam begins having an effect (as can be seen as well for other antibiotics in this group) which causes the heatflow to decrease after additional 300 min (Fig. 2A).

It was expected that ampicillin and piperacillin would show similar effects on the heatflow curves at subinhibitory concentrations. However, this was not the case (Fig. 2A). Although it was not possible to determine the MIC for ampicillin, one can see that 8 mg l^-1 ^ampicillin only decreased *P*_*max *_and had no effect on the detection time for bacterial activity, in contrast to piperacillin. It is an indication that *E. coli *metabolism reacts differently with each of the antibiotics. Further analysis of this difference was beyond the scope of this study.

Amikacin and gentamycin are both aminoglycosides acting on the 30S ribosome by inhibition of the translocation of the growing polypeptide chain from the A to the P site [20]. The same mode of action is clearly demonstrated in the profile of the IMC heatflow curves (Fig. 3A). There are only minor differences between the heatflow curves which may mostly reflect variations introduced by manual preparation of the samples. The heat curves, however, differ a bit more (Fig. 3B). This was most likely due to a reduced activity of the amikacin used as evidenced by finding an MIC above the recommendations of the CLSI [15]. It would be interesting to see whether antibiotics interacting with protein synthesis but with another site of action (like chloramphenicol on *S. aureus*) could also be differentiated as is the case for *S. aureus *(see above).

## Conclusion

We were able to show that isothermal microcalorimetry could be a powerful tool for MIC determination of antibiotics for any cultivable bacterium. There was no time saving possible since MICs were based on the conventional approach – evidence of growth at 24 hours. However, it is clear that determining MICs by IMC has the added advantage of allowing detailed comparative evaluation of the effects of subinhibitory antibiotic concentrations on growth-related thermodynamic activity of bacteria.

Furthermore, our study showed that the results are in agreement with the tests performed with a standard method by CLSI (broth dilution method). We summarized the results in Table 1 to provide an easy comparison with the addition *t*_*delay *_and *P*_*max *_of one concentration below the MIC to show how calorimetry data indicate the mode of bacterial action.

It might be possible to use an IMC approach to reduce the time for MIC determinations. For example, one might be able to develop a method to analyze the first few hours of IMC data for a series of antibiotic concentrations mathematically and extrapolate the MIC value. Also, by knowing the dissociation constant of an antibiotic, it would be possible to quantitatively characterize the inhibitory effect using the methods described in the study of Antoce *et al*. [11]. This might allow help extrapolation to the MIC value for a given antibiotic.

It seems likely that IMC studies of the type described here could be useful in antibiotic research and development. Early-stage IMC "screening" evaluations could help evaluate the mode of action of any new antimicrobial compound through comparison with known IMC profiles for the same type and strain of microorganism. The efficacy of compound modifications could be quickly screened by comparing new results with those for earlier formulations.

IMC studies of bacterial activity may also be of use in assessing the effects of phenotypic, genomic and proteomic modifications of microorganisms [23].

Overall, IMC has great power for microorganism activity studies, due to its high reproducibility and ability for simultaneous independent, quantitative evaluation of multiple samples at a given common temperature (e.g. 48 samples in the instrument used). Also, since IMC is completely passive, specimens are undisturbed, and after any period of IMC measurement, the ampoule contents (media, bacteria, etc.) can be analyzed by any other method desired. Finally, the continuous IMC data are amenable to mathematical treatment, and the IMC technique generally lends itself to future automation.

## Methods

### Isothermal microcalorimetry (IMC)

A TAM 48 (Thermal Activity Monitor 48, TA Instruments, Lukens Drive, New Castle, DE) was used. This instrument is designed for parallel multi-sample experiments with 4 ml ampoules. It is comprised of a thermostat containing 48 separate calorimeters which the thermostat maintains at a selected constant temperature. The individual calorimeters each have a dynamic range ± 50 mW, the short-term noise is less than ± 100 nW, the baseline drift/24 h is less than ± 200 nW.

In this study 4 ml ampoules were filled with 2.97 ml of growth media containing either no antibiotic or a known amount (details below) plus 0.030 ml of a bacterial inoculum (details below). Each ampoule was sealed from the environment and put individually into one of the 48 calorimeters, which were already equilibrated at 37°C and maintained at 37°C by the thermostat's control system. The ampoule insertion process transiently disturbs the equilibration, and thus useful heat flow rate data were not obtained for the first ~60 minutes (details below). Heat flow was sampled at rate of 1 Hz in J/s or W. Optionally, the heat flow rate vs. time data file can be exported for further evaluation, e.g. calculation of total energy in J produced in time *t*, compared to baseline.

### Bacterial strains and growth medium

The strains used in this study were the reference strains for MIC determinations as recommended by the CLSI manual [15]. *Escherichia coli *ATCC25922 was grown on LB agar plates or broth (Difco, Chemie Brunschwig, Basel, Switzerland) and *Staphylococcus aureus *ATCC29213 was cultivated on BHI agar plates or broth (Difco). The cultures were kept at -80°C in their respective growth media supplemented with 30% glycerol (Fluka, Buchs, Switzerland). Prior to use for the MIC determinations, they were cultivated on agar plates as recommended by the CLSI [2].

### Inoculum preparation

The inoculum preparation was as follows: A single colony was picked from a fresh plate and incubated overnight at 37°C in the respective growth medium. This culture was then adjusted with 0.01 M phosphate buffered saline pH 7.4 (PBS, Lab Dr. Bichsel, Interlaken, Switzerland) to an OD_600 _of 0.01.

### Antibiotics preparation

The 12 antibiotics used in this study for *E. coli *and *S. aureus *were chosen from among those listed in the CLSI manual [15]. All antibiotics were purchased from Fluka, Buchs, Switzerland. The required concentrations were prepared in cation-adjusted Mueller-Hinton Broth (MHII, Mueller Hinton II broth, Difco) by serial dilution from a stock solution according to the CLSI manual [15]. The Results section indicates which antibiotics were evaluated with which bacteria and at what concentrations.

### Sample preparation for microcalorimetry

Prior to use, the ampoules and the closures (rubber septa with integrated metal crimp-seal collars) were washed and separately sterilized (121°C, 20 min). They were then aseptically filled with 2.97 ml of MHII with or without added antibiotic and inoculated with 1% (30 μl) of the prepared inoculum (as described above). In addition, blanks were prepared (media alone, no inoculum) and evaluated calorimetrically to verify that measured heat flows were in all cases due only to microbial activity.

Prior to inserting ampoules, the thermostat and its calorimeters were equilibrated for at least 45 min at 37°C. The ampoules were then inserted in the calorimeters and lowered into the equilibration position. (Each of the 48 calorimeters is a separate instrument, and each evaluation is started, recorded and stopped separately.) At 15 min post-insertion, the ampoules were lowered down to the measuring positions. Then, 45 min later, after a calorimeter's heat flow signal has regained stability, the actual measurement of the heatflow vs. time started. This time was taken as time zero for the evaluation of the data and was thus actually ~1 hour after introducing the inoculum into the medium at room temperature.

### Standard interpretation method

Unless otherwise stated, each standard (non-calorimetric) experiment was performed in parallel with a calorimeter ampoule placed in a water bath at 37°C and evaluated after 24 h incubation using a photometer set at a wavelength of 600 nm. The sample preparation and the ampoules used for these experiments were the same as for the IMC experiments. All experiments, IMC and standard method, were performed in triplicate.

## Authors' contributions

AUD, DW and UVA conceived the study. UVA performed the experiments and wrote the manuscript. AUD, DW and UVA evaluated the results. AUD and DW revised the manuscript. All authors read and agreed to the manuscript.
